# Validation of a Parkinson Disease Predictive Model in a Population-Based Study

**DOI:** 10.1155/2020/2857608

**Published:** 2020-02-21

**Authors:** Irene M. Faust, Brad A. Racette, Susan Searles Nielsen

**Affiliations:** ^1^Washington University School of Medicine, Department of Neurology, St. Louis, Missouri, USA; ^2^University of the Witwatersrand, School of Public Health, Faculty of Health Sciences, Johannesburg, South Africa

## Abstract

Parkinson disease (PD) has a relatively long prodromal period that may permit early identification to reduce diagnostic testing for other conditions when patients are simply presenting with early PD symptoms, as well as to reduce morbidity from fall-related trauma. Earlier identification also could prove critical to the development of neuroprotective therapies. We previously developed a PD predictive model using demographic and Medicare claims data in a population-based case-control study. The area under the receiver-operating characteristic curve (AUC) indicated good performance. We sought to further validate this PD predictive model. In a randomly selected, population-based cohort of 115,492 Medicare beneficiaries aged 66–90 and without PD in 2009, we applied the predictive model to claims data from the prior five years to estimate the probability of future PD diagnosis. During five years of follow-up, we used 2010–2014 Medicare data to determine PD and vital status and then Cox regression to investigate whether PD probability at baseline was associated with time to PD diagnosis. Within a nested case-control sample, we calculated the AUC, sensitivity, and specificity. A total of 2,326 beneficiaries developed PD. Probability of PD was associated with time to PD diagnosis (*p* < 0.001, hazard ratio = 13.5, 95% confidence interval (CI) 10.6–17.3 for the highest vs. lowest decile of probability). The AUC was 83.3% (95% CI 82.5%–84.1%). At the cut point that balanced sensitivity and specificity, sensitivity was 76.7% and specificity was 76.2%. In an independent sample of additional Medicare beneficiaries, we again applied the model and observed good performance (AUC = 82.2%, 95% CI 81.1%–83.3%). Administrative claims data can facilitate PD identification within Medicare and Medicare-aged samples.

## 1. Introduction

Parkinson disease (PD) is a progressive neurodegenerative disorder that is diagnosed on the basis of motor symptoms. However, PD is characterized by both motor and nonmotor symptoms, which evolve over years. The period preceding PD diagnosis in which these symptoms occur, called the prodromal period, may predate the diagnosis by decades [1]. Traumatic brain injuries [2], fractures [3, 4], and injurious falls more generally [4] occur more frequently among individuals with undiagnosed PD relative to comparable individuals without PD. Motor symptoms occur for at least five years prior to PD diagnosis [5], and the increased risk of fractures among prodromal PD patients begins ∼6-7 years prior to PD diagnosis [3]. Accordingly, tools to facilitate early PD diagnosis could have both broad application and potentially important public health impact, for example, through a reduction in fall-related morbidity [2–7]. Since the motor symptoms are primarily due to loss of dopaminergic neurons in the substantia nigra, motor symptoms during the prodromal period are likely to be responsive to dopaminergic therapy.

Early identification of PD might be facilitated by known strong associations between PD and age, sex, race/ethnicity [8], smoking [9, 10], and several medical conditions including anosmia/hyposmia [11], constipation [12, 13], REM sleep behavior disorder [14], and depression [15, 16]. These are typically included as core predictors in algorithms for identifying PD, and we recently identified numerous other predictors in a cross-validated model based only on Medicare data using a population-based sample of beneficiaries with and without incident PD [17]. Within this same case-control sample, this model performed well, with an area under the receiver-operating characteristic curve (AUC) of 85.7%. A predictive model that only uses administrative health care data can potentially be applied to screen the entire population. Therefore, we sought to further evaluate the performance of our predictive model by reapplying the model. We applied the model to controls from our original study and then followed them for five years for PD diagnosis. In addition, we evaluated model performance by reapplying the model in an even larger, independent random sample of Medicare beneficiaries, in order to demonstrate external validity.

## 2. Materials and Methods

### 2.1. Study Approval and Data Protection

The Washington University Human Research Protection Office and the Centers for Medicare and Medicaid Services (CMS) approved the study. All data were deidentified prior to release from CMS.

### 2.2. Study Overview

**Figure 1:**
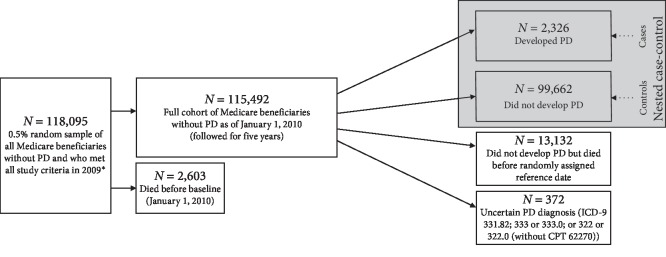
Participants in the cohort and nested case-control sample (U.S. Medicare). ^∗^Selection of participants in both the cohort and the nested case-control sample was according to the following criteria: all beneficiaries were required to have Medicare Part A and/or B coverage, be U.S. residence, be of age 66–90 in 2009, and be alive without PD as of January 1, 2010. We followed participants from January 1, 2010, through December 31, 2014. 118,095 beneficiaries did not have PD in 2009 and served as the controls in the original case-control study in which the PD predictive model was developed [17]. Abbreviations: CPT = Current Procedural Terminology; ICD-9 = International Classification of Diseases, Version 9; PD = Parkinson disease.

We used U.S. Medicare data to conduct all analyses. Nearly all individuals ≥65 years old in the U.S. utilize Medicare, thus allowing for the construction of large, nationwide, population-based studies in this age group. In the present work, we started with all controls from our original PD case-control study [17], who represented a population-based sample of beneficiaries age eligible for Medicare and without PD in 2009 (Figure 1). We then restricted to those who survived through the end of 2009 and followed this cohort for up to five years. From this cohort, we constructed a nested case-control sample to formally attempt an internal validation of the predictive model. The cohort and nested case-control sample, as well as the specific Medicare data, study criteria, and case ascertainment method that we used to construct them, are detailed below.

### 2.3. Available Medicare Data

We used Medicare data from 2004 to 2014. We used both base files and detailed claims data. Base files enumerate all beneficiaries in the respective year and provide basic demographic information and a summary of insurance coverage for each beneficiary. We used the base file from 2009 to assess study eligibility criteria and to identify the above cohort. We used base files from 2010 to 2014 to follow this cohort forward, i.e., ascertain vital status and date of death if applicable. We used carrier (physician/supplier part B), outpatient, inpatient, skilled nursing facility, durable medical equipment, and home health care claims data from 2004 to 2014. Claims files include the International Classification of Diseases, Ninth Revision (ICD-9) diagnosis, ICD-9 procedure, Current Procedural Terminology (CPT) procedure, and other Healthcare Common Procedure Coding System (HCPCS) codes. We used these files to determine the PD status and date of diagnosis and all medical predictor variables including smoking.

### 2.4. PD Ascertainment Method

We identified PD as ≥1 ICD-9 332 or 332.0 code, without Lewy body dementia (ICD-9 331.82), other extrapyramidal disease and abnormal movement disorders (ICD-9 333 or 333.0), or typographic error (nonpyogenic meningitis (ICD-9 322 or 322.0) without diagnostic lumbar puncture (CPT 62270)) [17]. We considered the date of the first code as the date of diagnosis. We noted whether any codes for PD were from a neurologist.

### 2.5. Study Participants and Follow-Up Period

We derived the cohort and nested case-control sample from the original controls in the case-control study we used to develop the PD predictive model validated here [17]. These original controls represented a 0.5% random sample of all Medicare beneficiaries who met all of the following criteria in 2009: no PD, age 66–90, U.S. residence, and Medicare Part A/B coverage but no non-Medicare coverage. We restricted to beneficiaries who were at least 66 years, 11 months old to ensure that all beneficiaries represented a population-based sample and had at least two full years of claims data. We restricted to beneficiaries aged ≤90 years because it is well documented that age-specific incidence tapers in older age groups, especially after ∼85–90 years of age [8, 17, 18]. The cohort in the present work comprised all of the original controls who were alive at the beginning of follow-up on January 1, 2010 (*N* = 115,492) (Figure 1). We followed this population-based cohort for up to five years (until PD diagnosis, death, or end of follow-up on December 31, 2014, for a mean follow-up time of 4.4 years and standard deviation (SD) of 1.3 years).

Within this cohort, we constructed a nested case-control study. Cases were all cohort members who developed PD in the five years of follow-up (*N* = 2,326 incident PD cases), and controls were all noncases who survived to a randomly assigned reference date during the same period (*N* = 99,662) (Figure 1). This nested case-control sample paralleled the original case-control study [17], which allowed us to recalculate the predictor variables, and hence probability of PD, as of the PD diagnosis date or comparable reference date.

### 2.6. PD Predictive Model in the Original Case-Control Study

We published our PD predictive model in detail previously [17]. This model includes the following predictors: age, sex, race/ethnicity, ever smoking, the total number of unique (distinct) diagnosis codes, and each of 536 individual diagnosis or procedure codes. Categories with >10 codes that positively predicted PD included PD symptoms, autonomic symptoms, trauma/falls, gait/balance, immobility, psychiatric conditions, cognitive conditions, and tests designed to diagnose medical conditions other than PD (e.g., brain imaging and blood tests for diabetes, hypothyroidism, vitamin B deficiencies, and syphilis). Prominent categories of diagnosis and procedure codes that were negative predictors of PD related to physical activity, cardiovascular disease, cancer, infectious conditions, and tobacco use. Of the 536 codes in the predictive model, 54 (10.1%) were HCPCS codes, which are more specific to Medicare than ICD-9/CPT codes because HCPCS codes are not universally used. These 54 HCPCS codes included medical transportation, durable medical equipment, nonoral drugs such as chemotherapy, and orthotic and prosthetic procedures.

### 2.7. Calculation of Predictors and the Probability of PD

We applied the above PD predictive model in the present study, hereafter the “full” model. Specifically, to estimate the predicted probability of PD for each participant in the present study, we (1) determined the value of each predictor (e.g., presence or absence of each diagnosis/procedure code), (2) multiplied each predictor by the respective coefficient that we previously published [17], (3) summed the resulting products, and (4) added the original model's constant term. We used the 2009 base file to calculate age and to obtain sex and race/ethnicity. We used these same demographic data and >600 diagnosis/procedure codes from the claims data to estimate the probability of having ever smoked tobacco [17, 19]. We assigned beneficiaries with a tobacco-specific code (e.g., ICD-9 V15.82 or 305.1) a smoking probability of 100% and then used a validated logistic regression predictive model to assign smoking probability to all other beneficiaries. Finally, we used the claims data to determine the total number of unique diagnosis codes and the presence or absence of each of the 536 diagnosis/procedure codes in the predictive model. We considered all codes from 2004–2014 up to the PD diagnosis or control reference date when recalculating the predicted probability of PD for the nested case-control sample to parallel the original case-control study. Secondarily, we restricted to codes from 2004 to 2009 when calculating the probability of PD as of baseline (January 1, 2010). This restriction had the practical effect of applying an exposure lag of up to five years (mean of 2.4 years in the nested case-control sample). Therefore, we only applied this restriction when necessary, i.e., for the cohort or to examine the effect of lagging in the nested case-control sample.

For comparison to the “full” model, we repeated the above for a simpler model, henceforth the “basic” model. In this basic model, we calculated the probability of PD using only age, sex, race/ethnicity, smoking, constipation, REM sleep behavior disorder, and anosmia/hyposmia as predictors, either with or without the total number of unique diagnosis codes. We also calculated the probability of PD on the basis of age alone, henceforth the “age-only” model.

### 2.8. Statistical Analysis

We used Stata [20] for all analyses. We calculated the overall PD incidence for this population-based sample of Medicare-aged individuals by dividing the total number of new PD diagnoses (2,326) by the total number of person-years at risk in the cohort over the five years of follow-up (504,246). We calculated person-years directly using the PD date of diagnosis in the claims files and the date of death from the base files. We estimated the relative risk of PD in relation to selected demographic characteristics by calculating odds ratios (ORs) and 95% confidence intervals (CIs), using logistic regression, in the nested case-control sample. We established a two-sided alpha (*α*) of 0.05 as the cutoff level for statistical significance.

To assess performance of the PD predictive model, we calculated the following recommended [21] metrics in the nested case-control sample: (1) difference between the mean predicted probability of PD for those who did and did not develop PD; (2) AUC; and (3) sensitivity and specificity. We calculated the latter at the cut point that balanced sensitivity and specificity, rather than the cut point that maximized the number classified accurately, because most participants did not have PD. We repeated these calculations in secondary analyses in which we either (1) restricted cases to 449 (19%) who had ≥1 PD diagnosis code from a neurologist, (2) restricted cases to 1,596 (69%) who had ≥2 PD diagnosis codes, (3) used our secondary (lagged) predictors, or (4) eliminated the contribution of the 54 HCPCS codes (assumed a value of zero). We included the latter in order to investigate the transportability of the predictive model to non-Medicare administrative claims data in which only ICD-9 and CPT codes are available. In order to investigate the external validity of the predictive model to samples that are demographically different from our U.S. population-based sample, we also repeated analyses while stratifying by age, sex, and race/ethnicity.

Finally, in the cohort, we conducted a survival analysis to assess the association between the predicted probability of PD at baseline and the development of PD. In this survival analysis, we followed all participants from January 1, 2010, through the date of PD diagnosis, death, or December 31, 2014, whichever came first. We censored beneficiaries with a diagnosis of Lewy body dementia, other neurodegenerative disease of the basal ganglia, or probable typographic error at the first date of the excluded diagnosis; that is, these individuals were not counted as having developed PD in survival analysis. We tested the proportional hazards assumption using Schoenfeld residuals. We then used Cox proportional hazards regression to calculate the hazard ratio (HR) and 95% CI for PD, i.e., the time to PD diagnosis, in relation to the predicted probability of PD. We categorized the probability in deciles to allow for a nonlinear association.

## 3. Results

### 3.1. PD Incidence and Demographic Risk Factors

The average annual incidence of PD in our population-based sample of Medicare-aged beneficiaries over five years was 461 per 100,000 person-years. The mean time to the first diagnosis code with PD was 2.37 years (standard deviation (SD) 1.4, median 2.29) after baseline. The risk of PD was greater with increasing age, for men, in non-Hispanic white than black individuals, and among never smokers (Table 1).

### 3.2. Predictive Model Performance: Primary Analysis

When we applied the full model to the nested case-control sample, the AUC was 83.3% (95% CI 82.5%–84.1%), fairly similar to our previous estimate. Sensitivity was 76.7% (95% CI 76.4%–77.0%) and specificity was 76.2% (95% CI 76.0%–76.5%) at the cut point that most closely balanced sensitivity and specificity. Likewise, 76.2% of participants were classified correctly at this cut point. The difference between the mean predicted probabilities of cases and controls was 0.33.

### 3.3. Predictive Model Performance: Secondary Analyses

All of the above measures were the same or slightly better when we restricted to cases with ≥1 code for PD from a neurologist (AUC = 83.6%, 95% CI 81.8%–85.4%) or to cases with ≥2 codes for PD (AUC = 83.9%, 95% CI 82.9%–84.8%). When we eliminated the contribution of the 54 HCPCS codes, model performance was not materially reduced (AUC = 83.3%, 95% CI 82.4%–84.0%; sensitivity 76.4% and specificity 76.2%). The AUC did not differ markedly according to sex (AUC = 82.7% in men, 83.6% in women) or race/ethnicity (83.5% in non-Hispanic white beneficiaries, 82.8% in black beneficiaries, and 80.8% in all other beneficiaries), but the AUC consistently decreased with age.

In the secondary analysis in which we restricted to diagnosis and procedure codes recorded prior to the baseline date on January 1, 2010, i.e., lagged the predictor variables, performance was lower. The AUC was 73.1% (95% CI 72.1%–74.2%). Sensitivity was 67.3% (95% CI 67.0%–67.6%) and specificity was 66.8% (95% CI 66.5%–67.1%) at the cut point that most closely balanced sensitivity and specificity. However, performance measures successively improved when we explored the effect of reducing the number of years of follow-up (mean lag).

**Figure 2:**
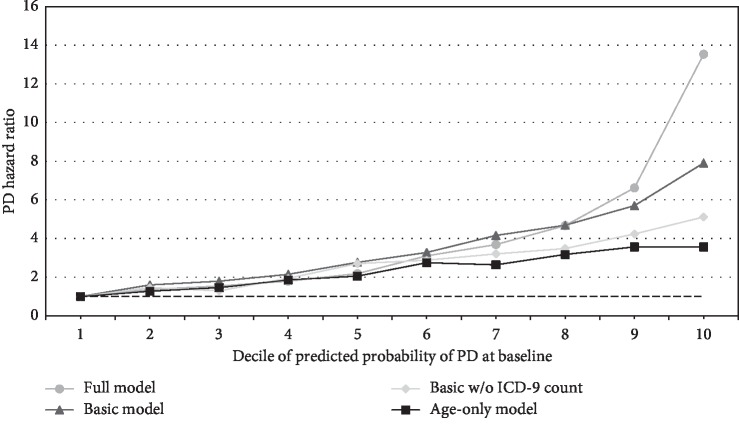
Hazard ratio of PD based on the predicted probability of PD at baseline (U.S. Medicare). Age-only model: predicted by age (2 linear splines with a knot at age 85). Basic model: predicted by age (2 linear splines with a knot at age 85), sex, race/ethnicity (7 categories), probability of ever/never smoking (continuous), constipation (ICD-9 564, 564.0, 564.00, 564.01, 564.02, and 564.09), REM sleep behavior disorder (ICD-9 327.42), and anosmia/hyposmia (included in ICD-9 781.1 as smell and taste disturbances), with or without the total number of unique ICD-9 diagnosis codes (continuous) as a measure of overall use of medical care. Full model: predicted by age, sex, race/ethnicity, smoking, total number of unique ICD-9 diagnosis codes, all as modeled above, and 536 diagnosis or procedure codes including codes for constipation and REM sleep behavior as above. Abbreviations: CI = confidence interval; ICD-9 = International Classification of Diseases, Version 9; HR = hazard ratio; PD = Parkinson disease. The predicted probability of PD as of baseline for the full model and both basic models was positively associated with the hazard ratio (HR) for PD (*p* < 0.001), with the highest HR occurring for the top decile of predictive probabilities from the full model (HR = 13.5, 95% CI 10.6–17.3). The predicted probability of PD for the full model departed from the three simpler models in the top 2-3 deciles of predicted probabilities when these probabilities were assessed in terms of the magnitude of their association with time to PD diagnosis.

The predicted probability of PD calculated as of baseline using the full model was strongly and significantly associated with time to PD diagnosis, i.e., the HR for PD, in the cohort (*p* < 0.001, Figure 2). The HR for PD among those with the top vs. the bottom decile of predicted probability of PD was 13.5 (95% CI 10.6–17.3). The full model performed better than the basic models and the age-only model at the top 2-3 deciles of predicted probability of PD.

### 3.4. Predictive Model Performance: *Post Hoc* Analysis

Given the above promising results, we sought to confirm external validity using an independent Medicare dataset in a *post hoc* analysis. Specifically, we obtained complete Medicare claims data from 2010 to 2014 from a random sample of Medicare beneficiaries who met the same eligibility criteria in 2009 as the original and above samples. We then focused on those who survived up to 2014 without the diagnosis of PD (*N* = 323,065; age 71–94 years, mean 79.6 years in 2014). Of these, we identified 1,365 with incident PD in 2014. We applied the predictive model to the claims data from 2010 to 2014 up to the PD diagnosis or random reference date to calculate the predicted probability of PD. The AUC was 82.2% (95% CI 81.1%–83.3%). Sensitivity was 75.8% (95% CI 75.7%–76.0%) and specificity was 76.7% (95% CI 76.6%–76.9%) at the cut point that most closely balanced sensitivity and specificity.

## 4. Discussion

In two large, population-based samples of Medicare beneficiaries, we validated the predictive model of PD that we developed previously [17]. Performance according to the AUC was similar to our earlier estimates, and, more importantly, the current validation study confirms that our PD predictive model is a possible cost-effective strategy to identify those with a relatively high probability of having prodromal PD. Earlier identification of PD could have important public health impact. First, earlier identification of individuals with prodromal PD may reduce morbidity and mortality through early treatment, given the growing evidence that prodromal PD patients have a greater risk of fall-related traumatic injuries than the general population [2–4]. Second, earlier PD identification would likely reduce the amount of diagnostic testing that prodromal PD patients often undergo as a consequence of the symptoms of undiagnosed PD. Third, clinical trials of potential neuroprotective therapies might have greater chance of demonstrating effectiveness when PD patients are treated prior to substantial loss of substantia nigra neurons. While we completed all model development and validation within U.S. Medicare-based samples, we confirmed that this model likely would be transportable to other administrative datasets as well, especially those with ICD-9 and CPT codes.

Ideally, a PD predictive model based on administrative data would identify people with prodromal PD using medical diagnosis codes that capture symptoms other than the early motor symptoms of PD. The motor symptoms generally begin occurring relatively close to diagnosis, so nonmotor symptoms are likely more important to the identification of PD even earlier. In this regard, we confirmed the feasibility of a useful predictive model based on lagged predictor variables. Even with our most aggressive lag, 2.4 years on average, the AUC indicated that the model performed substantially better than both chance and the simpler predictive models. Thus, future efforts should focus on developing a predictive model that identifies people with prodromal PD much earlier in their disease course. Whether these efforts will require additional data not readily available in medical claims data remains to be determined.

While these results are very encouraging and consistent with our initial validation of our PD predictive model, we do note some limitations. We conducted this further validation using a Medicare-based cohort, and we also used Medicare data to develop this predictive model. This cohort, however, was a large, randomly selected, population-based sample of individuals who are likely representative of individuals aged 65 years and older in the U.S. This model has yet to be applied to a younger or more racially diverse population or to a cohort that includes other types of insurance beneficiaries. Application in each of these groups is recommended to demonstrate the generalizability of the model. However, it is very encouraging that the performance of our model was particularly good in the youngest beneficiaries and did not differ markedly according to race/ethnicity. The relatively few HCPCS codes in the model potentially could be derived in non-Medicare claims data, or as we demonstrated, even excluded with little impact on model performance. All other diagnosis and procedure codes included in the model follow national (CPT) or international (ICD-9) classification systems for easy implementation in non-Medicare insurance claims in the U.S. and possibly some other countries as well. Another potential limitation is that both our cohort and nested case-control sample are not strictly independent of our original sample. Nonetheless, we also validated the predictive model in an independent Medicare cohort. Model performance again was good, only slightly lower, which is unsurprising given that this was an external validation in a somewhat older cohort. Finally, we relied on claims data for PD ascertainment. While the PD incidence we observed in the present population-based cohort is consistent with that in our earlier case-control study [17], it is markedly higher than that observed in another sample of individuals with insurance coverage [8]. This suggests that, in our study, some beneficiaries who received a code for PD might not have PD. Nonetheless, all metrics for validating the predictive model were improved, if anything, when we restricted PD diagnoses to those made by a neurologist or with ≥2 PD diagnosis codes.

## 5. Conclusion

In summary, our predictive model successfully predicted PD in both an internal and an external validation study. This predictive model has the potential to be used as a cost-effective means to facilitate the earlier identification of patients with PD by simply applying the model to medical claims data.

## Figures and Tables

**Table 1:** Characteristics of participants in the nested case-control study (U.S. Medicare).

	Cases	Controls	OR (95% CI)	Mutually adjusted
	*N* = 2,326	*N* = 99,662		
	*n* (%)	*n* (%)		OR (95% CI)^a^
*Age, years*				
66–69	189 (8.1)	16,456 (16.5)	1.0 (reference)	1.0 (reference)
70–74	552 (23.7)	31,739 (31.9)	1.51 (1.28–1.79)	1.51 (1.28–1.78)
75–79	623 (26.8)	23,202 (23.3)	2.34 (1.98–2.75)	2.29 (1.94–2.70)
80–84	572 (24.6)	17,481 (17.5)	2.85 (2.41–3.36)	2.75 (2.33–3.26)
85–90	390 (16.8)	10,784 (10.8)	3.15 (2.64–3.75)	3.05 (2.55–3.64)
Mean (SD)	78.0 (5.9)	75.9 (6.0)	1.058 (1.051–1.065)^b^	N/A
*Sex*				
Male	1,188 (51.1)	42,141 (42.3)	1.0 (reference)	1.0 (reference)
Female	1,138 (48.9)	57,521 (57.7)	0.70 (0.65–0.76)	0.57 (0.52–0.63)
*Race/ethnicity*				
White	2,056 (88.4)	85,941 (86.2)	1.0 (reference)	1.0 (reference)
Black	121 (5.2)	7,477 (7.5)	0.68 (0.56–0.81)	0.72 (0.60–0.87)
Pacific Islander/other^c^	25 (1.1)	1,715 (1.7)	0.61 (0.41–0.91)	0.65 (0.43–0.96)
Asian	57 (2.5)	2,257 (2.3)	1.06 (0.81–1.38)	1.02 (0.78–1.33)
Hispanic	56 (2.4)	1,878 (1.9)	1.25 (0.95–1.63)	1.16 (0.88–1.52)
Native American	11 (0.5)	394 (0.4)	1.17 (0.64–2.13)	1.26 (0.69–2.31)
*Smoking index* ^*d*^ *≥* *median*	1,172 (50.4)	56,100 (56.3)	0.79 (0.73–0.86)	0.72 (0.66–0.80)

OR = odds ratio; CI = confidence interval; SD standard deviation; N/A = not applicable. ^a^Mutually adjusted indicates that all ORs are adjusted for all other variables in the column. ^b^Per year of age. ^c^Includes 98 participants with race/ethnicity coded as “Unknown.” ^d^Predicted probability of ever smoking divided by the person's total number of unique diagnosis codes (or 1 for 5,804 participants without any diagnosis codes).

## Data Availability

Per our agreement with CMS, we are not allowed to share individual beneficiaries' data.
